# Precision of fibula positioning guide in mandibular reconstruction with a fibula graft

**DOI:** 10.1186/s13005-016-0104-2

**Published:** 2016-01-27

**Authors:** Se-Ho Lim, Moon-Key Kim, Sang-Hoon Kang

**Affiliations:** Department of Oral and Maxillofacial Surgery, National Health Insurance Service Ilsan Hospital, Goyang, Republic of Korea; Department of Oral and Maxillofacial Surgery, College of Dentistry, Yonsei University, Seoul, Republic of Korea

**Keywords:** Computer aided surgery, CAD/CAM, Surgical guide, Mandible reconstruction

## Abstract

**Background:**

This study examined the usefulness of the fibula positioning guide for boosting the accuracy of mandible reconstructions.

**Methods:**

Thirty mandibular rapid prototype (RP) models were allocated to experimental (*N* = 15) and control (*N* = 15) groups. For reference, we prepared a reconstructed mandibular RP model with a three-dimensional printer, based on surgical simulation. In the experimental group, a fibula positioning guide template and fibula cutting guide, based on simulation, were used to reconstruct the mandible with a fibula graft. In the control group, only the fibula cutting guide, with reference to the reconstructed RP mandible model, was used to reconstruct the mandible with a fibula graft. The two mandibular reconstructions were compared to the surgical simulation by registering images with the non-surgical right side of the mandible. On the reconstructed side, 3D measurements were compared between the surgical simulation and actual surgery, and the sum of differences was taken as the total error.

**Results:**

The combined use of the fibula cutting and positioning guides produced a smaller total error (mean ± SD: 10.0 ± 7.9 mm) than the fibula cutting guide alone (12.8 ± 8.8 mm; *p* = 0.015). The greatest point error was the vertical error at the mesial point of the anterior fibula segment. The anteroposterior and lateral errors were not significantly different between groups. These results showed that these two methods were not significantly different, except in the total and vertical errors.

**Conclusions:**

Considering the CAD/CAM processes required for creating positioning devices, the benefit provided with a positioning guide justified its use over the fibula cutting guide alone.

## Background

The wide distribution of computed tomography (CT) imaging services and advancements in computer technology in recent years have offered surgeons the ability to conduct preoperative surgical simulations. Surgical guides can be manufactured with the use of computer-aided design (CAD) and computer-aided manufacturing (CAM) technologies. These guides help surgeons adhere to surgical simulation plans in actual surgeries for craniofacial reconstructions [1, 2].

The free fibula flap is one of the most commonly-used grafts in mandibular reconstruction. It offers several advantages over other flaps, including a bone length sufficient for mandible reconstruction, a high survival rate, and attached skin for concurrent skin grafting [3]. Mandible reconstruction with a FFF is preceded by surgical simulation to determine the details of the mandibulectomy, which gives preoperative information on the number of fibula bone segments needed and the cutting angles. CAD/CAM techniques are used to manufacture surgical guides to assist in cutting the fibula, according to the preoperative simulation plans. The fibula cutting guide is prepared by converting CT information about the positions of the osteotomy lines and bone segment placements into stereolithography (STL) data. The guide is designed with a computer, based on data acquired in virtual surgical simulations; then, it is manufactured with a three-dimensional (3D) printer and biocompatible materials [4]. The fibula cutting guide facilitates cutting the fibula bone segments at the correct angles to ensure the segments fit together when placed into the mandible during reconstruction. Thus, using the guide enhances the reconstruction outcomes in the patient [5]. Unfortunately, however, positioning a fibula segment in the mandible is more difficult during an actual reconstruction surgery than it is in the surgical simulation. Methods for placing the fibula bone segments into mandibular reconstruction sites have been reported previously [6, 7]. Fibula segments can be fixed with plates of various sizes and materials, including metal reconstructive plates, mini plates, or resorbable plates. Moreover, a number of methods can be used to guide the fibula bone segments into the reconstructive sites, including methods involving computer imaging techniques (e.g., navigation) [8]. However, few reports have described the use of a fibula positioning guide designed to facilitate the correct placement of fibula segments during mandibular reconstructions.

In the present study, based on CT data, we conducted a surgical simulation of a mandibular reconstruction with a fibula graft. In addition, we used CAD/CAM techniques to manufacture a fibula cutting guide and a fibula positioning guide to facilitate cutting and placing the fibula segments into the correct location during mandible reconstruction, according to the surgical simulation. The results elucidated the usefulness of a fibula positioning guide in boosting the accuracy of mandible reconstructions.

## Methods

### Rapid prototype models of the mandible and fibula

**Fig. 1:**
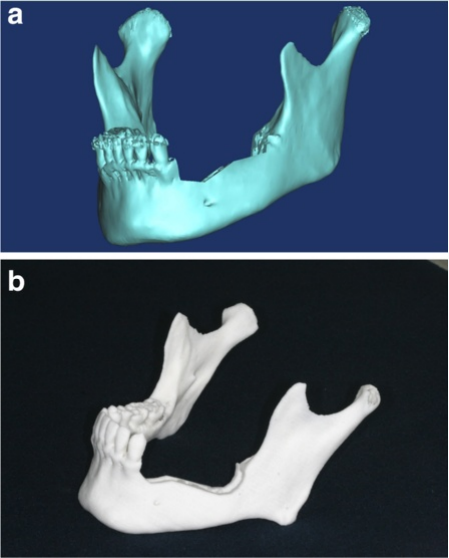
**a** 3-dimensionally (3D) reconstructed image of a mandible with a defect on the left side. **b** 3D printed RP mandible model with defect on the left side

Based on CT data of 15 mandibles, we prepared computer-assisted 3D-image reconstructions of mandibles with a defect on the left side (Fig. 1a). We then used stereolithographic (STL) data and a three-dimensional (3D) printer (ProJet 360, 3D Systems, Inc, Rock Hill, SC) to manufacture 15 pairs (*N* = 30) of rapid prototype (RP) models of mandibles with partial defects on the left side (Fig. 1b). Each pair of models was separated; one was assigned to the control group (*N* = 15) and the other to the experimental group (*N* = 15).

The CT data for one left fibula were used to prepare a computer-assisted 3D reconstructed image the fibula. Again, the STL data and the same 3D-printer (ProJet 360) were used to manufacture 30 RP models of the fibula. These were assigned to control and experimental groups (*N* = 15 models each).

### Surgical simulation for mandibular reconstruction

**Fig. 2:**
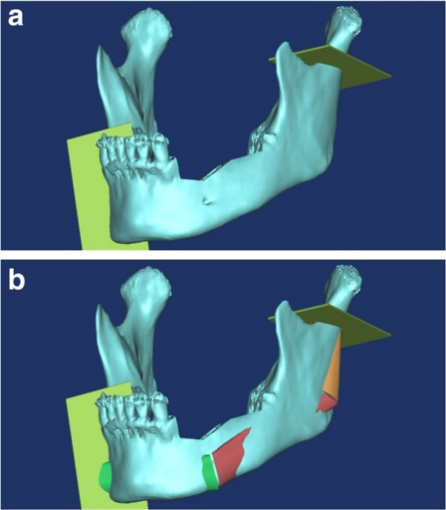
**a** Cuts performed in the mandible osteotomy. Plates show the cutting planes for removing the section from the right first premolar area to the left condyle neck in the surgical simulation. **b** Surgical simulation image of mandibular reconstruction with the fibula graft

**Fig. 3:**
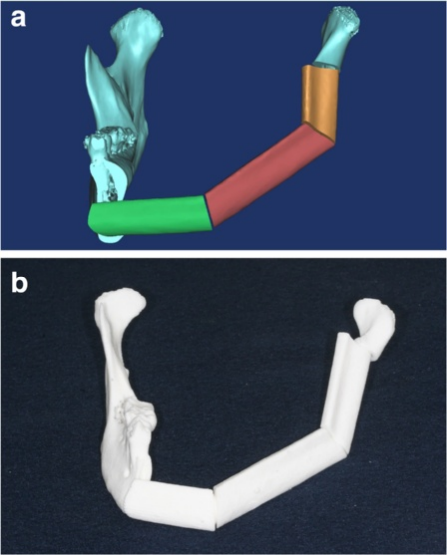
**a** 3D image of the mandible reconstructed with fibula segments in a surgical simulation. **b** 3D printed RP model of reconstructed mandible with fibula graft

The surgical simulation process for mandibular reconstruction was as follows: (1) We acquired CT DICOM data by scanning the mandible and fibula models (1.0 mm slices; Siemens Sensation 64 CT scanner, Siemens AG, Erlangen, Germany). (2) We opened the mandible and fibula DICOM files with Mimics version 14.0 software (Materialise, Leuven, Belgium) to convert them into 3D images. (3) To reconstruct the left mandible (defective area) in the simulation surgery, first we cut the mandible image from the right 1st premolar to the left inferior condylar neck area (Fig. 2a). (4) We placed the 3D fibula image on top of the mandibulectomized area created in the simulated surgery. (5) We then bent the fibula at the canine area and the mandibular angle area to make it fit the curves in the simulated mandible (Fig. 2b). This provided the lengths and angles of three fibula segments. We used the STL file of the left mandible reconstructed with the 3 fibula bony segments (Fig. 3a) and the ProJet 360 3D printer to manufacture the RP reconstructed model (Fig. 3b).

### Control group

**Fig. 4:**
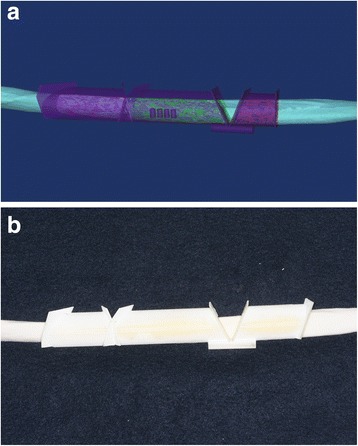
**a** Design of fibula cutting guide, based on surgical simulation of mandibular reconstruction. The image of the guide (*purple*) is superimposed on the image of the intact fibula. **b** 3D-printed fibula cutting guide placed on the intact RP fibula

We manufactured a fibula cutting guide to facilitate cutting the fibula according to the surgical simulation. First, we designed the fibula cutting guide in the Mimics software. We moved the fibula bone fragments that were used to reconstruct the left mandible to their original positions in the intact fibula bone. We rendered planes that would guide cutting, based on the cross sections of the fibula fragments (Fig. 4a). We used the STL data of the designed fibula cutting guide to manufacture the fibula cutting guide (Fig. 4b) with the 3D printer (ProJet 3500 HDMax 3D Printer, 3D Systems, Inc, Rock Hill, SC).

**Fig. 5:**
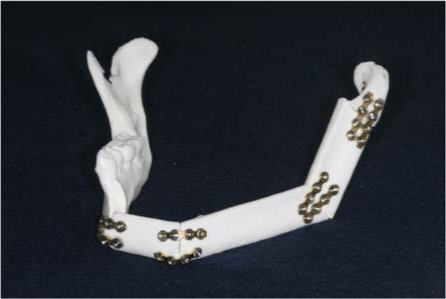
Experimental model of a mandible reconstructed using only the fibula cutting guide (control group)

For the surgery, we cut the fibula RP model according to the manufactured fibula cutting guide with a fissure bur, an industrial compass saw, and a surgical osteotome. We performed the osteotomy on the RP mandible according to the mandibular surgical simulation, by visually referring to the reconstructed RP model. We used an industrial-grade compass saw to remove the defective left mandibular area, as per the manual. The mandible was cut from the right anterior 1st premolar to the left condylar neck. The cut fibula bony segments were placed with reference to the reconstructed RP model. Then, titanium mini plates (Jaeil, Seoul, Korea) were bent to fix the fibula bony segments (Fig. 5), with two plates at each connection site.

### Experimental group

**Fig. 6:**
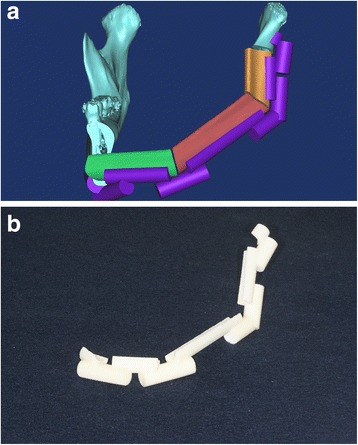
**a** Design of fibula positioning guide for the mandible reconstruction in a surgical simulation. **b** 3D-printed fibula positioning guide

To facilitate placing the fibula segments into the mandible, we designed a fibula positioning guide for each mandible in a reconstruction simulation. The positioning guide comprised five supports that fit onto the remaining right inferior border of the mandible, the three fibula segments, and the left condylar region. These components were stabilized into the correct positions by connecting them to supporting poles (Fig. 6a). Based on the STL data of this design, the fibula positioning guide was manufactured (Fig. 6b) with the 3D printer (ProJet 3500 HDMax 3D Printer, 3D Systems, Inc, Rock Hill, SC).

**Fig. 7:**
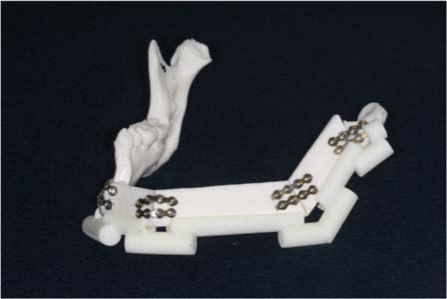
Experimental model of a mandible reconstructed using a fibula cutting guide and a fibula positioning guide (experimental group)

For the surgery, in the same manner as in the control group, we first cut the RP mandible, and then we cut the RP fibula model with the fibula cutting guide. Next, we placed the remaining regions of the mandible RP model and the cut RP fibula segments in the positioning guide. Finally, we fixed all the components in the same manner as described above for the control group (Fig. 7).

### Superimposed surgical simulation data and actual surgical data for error measurement

**Fig. 8:**
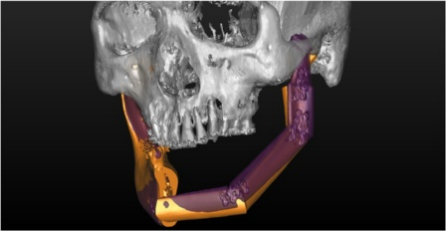
Superimposition of the surgical simulation image (*orange*) and the postoperative image (*purple*), registered to the right non-surgical mandibular areas to perform measurements of the error

We acquired CT images of the RP mandible models after reconstruction surgery. The images were acquired under the same conditions as those used before the experiment. We imported the DICOM file of reconstructed mandible model into the Mimics software to convert it to 3D images, and we exported it in the STL file format. We also exported the surgical simulation data to an STL file. With XOV2 software (INUS Technology, Seoul, Republic of Korea), we superimposed the actual surgical data onto the surgical simulation data and registered them based on the non-operated right mandibular region (Fig. 8).

### Reference planes for error measurement

**Fig. 9:**
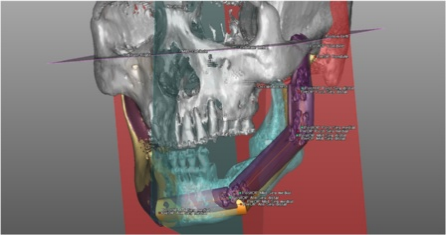
Reference planes, measurement points, and axes set to measure the errors in mandible reconstruction with fibula bony segments. Distances were measured from the reference planes (FH = *purple*; coronal = *red*; sagittal = *blue*) to the measurement points on the postoperative model (PostOP: *blue points*) and on the surgical simulation (PreOP: *red points*)

We opened the superimposed mandible data (surgical simulation and postoperative data) in the Mimics software. We compared the surgical result to the simulation by setting a reference plane in the cranial area and calculating the distances between the plane and several measurement points on the reconstructed mandibles. The error was taken as the difference between the measurements taken at corresponding points on the postoperative model and the surgical simulation. Three reference planes were established (Fig. 9): the horizontal plane (FH plane for vertical error), the mid-sagittal plane (sagittal plane for lateral error), and the coronal plane (for anteroposterior errors). The FH plane passed through the right orbitale (infraorbital margin) and the two porions (upper external auditory canal areas). The mid-sagittal plane was perpendicular to the FH plane and crossed the nasion and internal occipital crest. The coronal plane was perpendicular to both the FH plane and mid-sagittal plane, and passed through the nasion. Based on these three reference planes, three dimensional (3D) errors were calculated: the vertical error, the anteroposterior error, and the lateral error.

### Setting measurement points and measuring errors

Mandibles were measured at the lateral points of each fibula segment and the lateral pole of the left condyle (Fig. 9). One individual performed all the measurements at predetermined points that were set at identical locations in the surgical simulation images and the actual postsurgical model images. The vertical distances (Dv) were measured from the FH reference plane to the measurement point; the vertical error (Ev) was taken as the difference between the distance measured on the simulation (Dvs) and that measured on the postsurgical model (Dvm), as follows: Ev = Dvm–Dvs. The distances in the vertical (Dv), lateral (Dlat), and anteroposterior (Dap) directions were measured from the FH plane, mid-sagittal plane, and coronal plane, respectively, and the errors at each point were evaluated (Ev, Eap, and Elat, respectively). In addition, a line connecting the mesial and distal lateral points of each fibula segment was set as the axis of that segment. The differences in the angles formed between the axis in the simulation and the corresponding axis in the postsurgical model were measured. The shortest distance between corresponding measurement points on the simulation and postoperative model was considered a 3D distance error. The sum of the 3D distance errors was considered the total 3D error.

### Statistical analyses

We evaluated the errors in control and experimental groups to determine whether the fibula positioning guide offered any benefit in the mandibular reconstruction. We used a *t*-test to compare the measurements obtained with the two methods. The significance level was set to 0.05.

To measure the error in placing the measurement points by the surgeon, the surgeon repeatedly (10 times) set a reference point on the left condylar head area; the error was measured by calculating the differences in the reference points.

The error arising from performing the osteotomy without the use of a mandible cutting guide was computed by measuring the distances between the cut right mandibles in the surgery simulation and the postoperative model. The error in superimposing the mandibular images was analyzed by examining the total 3D error in the right mandible reference area.

## Results

First, we compared the 3D distance errors (distance between corresponding points on the surgical simulation and the surgical model) between the control and experimental groups (Table 1). When both the fibula cutting and positioning guides were used, the mean (±SD) total 3D distance error was smaller (10.0 ± 7.9 mm) than when only the fibula cutting guide was used (12.8 ± 8.8 mm; *p* = 0.015; Table 1). There was no significant difference between the two groups at any one measurement point, but all the measurement values were larger in the control group, when only the fibula cutting guide was used. The error increased for segments in the more posterior areas of each group; i.e., in both groups, the mean error was greater than 12 mm at all points posterior to the mesial point in the middle fibula segment. The error in the area from the middle fibula segments to the condylar head increased from 12 to 16 mm in the experimental group and from 16 to 19 mm in the control group.

**Table 1:** 3-d distance errors (mm) for the different surgical guide methods

Measurement point	Fibula cutting guide alone (control group)	Fibula cutting guide & fibula positioning guide (experimental group)	*P* value
Anterior segment-mesial point (*n* = 15)	4.3 ± 2.3	3.1 ± 1.5	0.096
Anterior segment-distal point (*n* = 15)	7.2 ± 3.2	5.7 ± 3.6	0.260
Middle segment-mesial point (*n* = 15)	7.7 ± 3.5	6.3 ± 3.6	0.290
Middle segment-distal point (*n* = 15)	16.5 ± 8.1	12.1 ± 7.7	0.147
Posterior segment-mesial point (*n* = 15)	16.8 ± 7.9	12.0 ± 7.9	0.111
Posterior segment-distal point (*n* = 15)	18.1 ± 9.8	14.5 ± 8.8	0.304
Condyle lateral point (*n* = 15)	19.4 ± 8.9	16.2 ± 8.7	0.338
Total (*N* = 105)	12.8 ± 8.8	10.0 ± 7.9	0.015*

Note: Values represent the mean ± standard deviation. The segment is the fibula segment used to reconstruct the mandible, and the position refers to its placement in the mandible. The condyle is on the left mandible, where it is articulates with the skull

*P < 0.05

We examined the vertical distances from the previously-established horizontal plane, and calculated the error differences between the surgical simulation and the postoperative data (Table 2). The mean (±SD) vertical distance error at the mesial point of the anterior fibula segment was −0.2 ± 1.1 mm in the experimental group and 1.8 ± 2.0 mm in the control group. Hence, the error was smaller when both the fibula cutting and positioning guides were used (*p* = 0.002). There were no significant differences between the groups at any other measurement point, except at the mesial point of the anterior fibula segment. The errors for segments posterior to the distal point on the middle fibula segment ranged from 2 to 5 mm in the experimental group and from 6 to 8 mm in the control group.

**Table 2:** Vertical distance errors (mm) for the different surgical guide methods

Measurement point	Fibula cutting guide alone (control group)	Fibula cutting guide & fibula positioning guide (experimental group)	*P* value
Anterior segment-mesial point	1.8 ± 2.0	−0.2 ± 1.1	0.002*
Anterior segment-distal point	−0.1 ± 5.6	0.1 ± 3.6	0.850
Middle segment-mesial point	−0.1 ± 6.1	0.1 ± 3.7	0.902
Middle segment-distal point	6.0 ± 12.3	2.8 ± 7.6	0.400
Posterior segment-mesial point	6.6 ± 12.3	3.6 ± 7.6	0.432
Posterior segment-distal point	8.5 ± 13.8	5.3 ± 8.7	0.460
Condyle lateral point	7.8 ± 12.9	3.5 ± 7.7	0.287

Note: Values represent the mean ± standard deviation. The segment is the fibula segment used to reconstruct the mandible, and the position refers to its placement in the mandible. The condyle is on the left mandible, where it is articulates with the skull. Negative values indicate that the position of the point in the model was closer to the FH plane than the corresponding point in the simulation

*P < 0.05

The overall mean anteroposterior errors (based on the coronal plane) were not significantly different between the two groups at each measurement point (Table 3). The error for the segments between the distal point on the middle fibula segment and the condylar head ranged from 3 to 4 mm in the experimental group and from −1 to 3 mm in the control group. With the exception of the error at the mesial point on the anterior fibula segment, all errors had positive values in the experimental group, which indicated an anterior displacement after the actual surgery.

**Table 3:** Anteroposterior errors (mm) for the different surgical guide methods

Measurement point	Fibula cutting guide alone (control group)	Fibula cutting guide & fibula positioning guide (experimental group)	*P* value
Anterior segment-mesial point	−2.0 ± 3.1	−1.8 ± 1.9	0.854
Anterior segment-distal point	−0.4 ± 5.0	1.0 ± 5.3	0.439
Middle segment-mesial point	−0.6 ± 5.2	0.6 ± 5.8	0.543
Middle segment-distal point	1.2 ± 10.4	4.3 ± 10.3	0.416
Posterior segment-mesial point	0.9 ± 10.6	4.1 ± 10.4	0.408
Posterior segment-distal point	−1.0 ± 11.6	3.0 ± 12.1	0.359
Condyle lateral point	3.2 ± 13.2	4.8 ± 13.9	0.738

Note: Values represent the mean ± standard deviation. The segment is the fibula segment used to reconstruct the mandible, and the position refers to its placement in the mandible. The condyle is on the left mandible, where it is articulates with the skull. Negative values indicate that the position of the point in the model was closer to the coronal plane than the corresponding point in the simulation

*P < 0.05

There were no significant differences between groups in the lateral errors (based on the sagittal plane) at each measurement point (Table 4). The groups had similar error values at the distal point of the anterior fibula segment (−1.7 mm) and at the mesial point of the middle fibula segment (−2 mm). Negative error values indicated a mesial displacement after surgery. In both groups, the errors in the segments between the middle fibula segment and the condylar head were positive, indicating a lateral displacement after the actual surgery.

**Table 4:** Lateral distance errors (mm) for the different surgical guide methods

Measurement point	Fibula cutting guide alone (control group)	Fibula cutting guide & fibula positioning guide (experimental group)	*P* value
Anterior segment-mesial point	0.4 ± 2.0	−0.8 ± 1.8	0.080
Anterior segment-distal point	−1.7 ± 2.3	−1.7 ± 1.6	0.997
Middle segment-mesial point	−2.1 ± 2.4	−2.0 ± 1.6	0.883
Middle segment-distal point	0.8 ± 7.3	2.4 ± 4.0	0.478
Posterior segment-mesial point	0.7 ± 7.1	1.7 ± 3.7	0.616
Posterior segment-distal point	4.5 ± 4.6	3.8 ± 5.0	0.694
Condyle lateral point	1.8 ± 6.6	1.6 ± 6.7	0.917

Note: Values represent the mean ± standard deviation. The segment is the fibula segment used to reconstruct the mandible, and the position refers to its placement in the mandible. The condyle is on the left mandible, where it is articulates with the skull. Negative values indicate that the position of the point in the model was closer to the sagittal plane than the corresponding point in the simulation

*P < 0.05

We measured the linear angle of each segment axis compared to the corresponding axis in the simulation, and found no significant angular errors with either method (Table 5). There was an error of about 12° in the posterior segment axis in the control group; but all other errors were under 10°. In the experimental group, errors ranged from 7.8 to 9.1°.

**Table 5:** Axis angles errors (°) for the different surgical guide methods

Measurement line	Fibula cutting guide alone (control group)	Fibula cutting guide & fibula positioning guide (experimental group)	*P* value
Anterior segment axis	9.9 ± 5.8	8.1 ± 5.3	0.375
Middle segment axis	9.5 ± 4.6	7.8 ± 4.1	0.285
Posterior segment axis	12.8 ± 8.1	9.1 ± 6.8	0.195

Note: Values represent the mean ± standard deviation

*P < 0.05

We also calculated the error in cutting the mandible by examining the differences between the surgical simulation and postoperative data. The mean (±SD) total 3D error in the right mandible cut was 1.1 ± 0.6 mm in the experimental group and 0.8 ± 0.5 mm in the control group; thus, the errors were not significantly different between the two groups (*p* = 0.138).

The mean (±SD) error arising from setting the measurement points by the surgeon was found to be 0.2 ± 0.1 mm.

The mean (±SD) total error in the superimposition of the reference areas (non-surgical right mandible area) of the model and simulation images was 0.009 ± 0.601 mm.

## Discussion

This study examined the precision of a fibula positioning guide in mandibular reconstruction with the fibula graft. We found significant errors between reconstructions performed with or without the positioning guide, in the mean total 3D distance and the vertical error at the mesial point in the anterior fibula segment.

In both groups, the 3D distance errors increased from the anterior to the posterior segments. At the points posterior to the middle fibula segment, which are considered the posterior mandible area, both groups showed a mean error greater than 12 mm. When only a fibula cutting guide was used, the error in the condylar head area was about 19 mm. In the present study, the position of the condyle was not fixed; instead, it was held by hand in position within the glenoid fossa to form the temporomandibular joint. Thus, the largest error was observed in the condylar area. This error was believed to be similar to the error associated with movement of the condylar head in the temporomandibular joint. This observation indicated that the condylar head is prone to displaying the greatest error in actual clinical cases.

When only the cutting guide was used, the mean vertical errors in the posterior mandible area were greater than 6 mm. When both the fibula cutting guide and the positioning guide were used, the mean vertical errors for the same region ranged from 2 to 5 mm. This pattern was similar to that observed in the 3D distance errors, and it indicates that the potential for error increased in the posterior direction. Consequently, in actual clinical cases, we can expect a greater potential for error in positioning segments in the posterior mandible.

There were no significant intergroup differences in the anteroposterior error at each measurement point. When a fibula positioning guide was used in the experimental group, the errors were somewhat higher than control group errors at points between the middle fibula and the condylar head areas (range 3 to 4 mm). On the other hand, when only the fibula cutting guide was used (control group), the errors in all measurement areas were ≤3 mm. This suggested that, in real clinical cases, it might be more beneficial to adjust the anteroposterior position of the fibula fragments by referring to the 3D-printed reconstructed model, rather than using a positioning guide.

There were no significant differences in lateral errors between groups at each of the measurement points. In both groups, the errors for the distal point of the anterior fibula segment (−1.7 mm) and the mesial point of the middle fibula segment (−2 mm) were similar in magnitude. The negative values indicated a medial displacement after surgery. Moreover, in both groups, the errors for the segments between the mid fibula segment and the condylar head were positive, indicating a lateral displacement after surgery. Therefore, in real clinical cases, the segments may change from a medial displacement in the anterior mandible to a lateral displacement in the posterior mandible, indicating an increasing lateral displacement.

There were no significant angular errors in any segments with either method. When only the fibula cutting guide was used, the error was approximately 9° for the anterior and mid fibula segments, and approximate 12° for the posterior segment. When the positioning guide was used, the error ranged from around 7 to 9°. The results of this experiment implied that, in real clinical cases, there may be an axis error of about 10° in each fibula segment.

A mandibular cutting guide may be required for mandibular osteotomy. In the present study, the average error in the right mandible area was about 1.1 mm; the same error was 0.8 mm in the control group. This result may imply that a fibula cutting guide is not required to enhance the precision of the mandibular osteotomy in an actual surgery.

Severe soft tissue damage may accompany mandibular reconstruction. In such cases, the surgeon must consider facial soft tissue in addition to reconstructing the mandible using a fibula. However, the present study only examined whether the fibula bone segment can be placed in the appropriate position, in accordance with the simulation surgery, in mandibular reconstruction. In other words, this study is limited to the reconstruction of hard tissue. Studies with actual clinical cases using fibula positioning devices are needed to examine facial reconstruction involving the fibula and soft tissues.

In this study, the three-dimensional distance error was significantly small when the positioning device was used. There was no statistically significant difference in the anteroposterior error, lateral error, and vertical error from each plane. However, the errors were smaller in general when the positioning device was used. This may be a statistically significant outcome, depending on the numbers included in the experimental and control groups. This study had 15 models for each group, and in such cases, the effect size is 0.94 when α (significance level) is 0.05 and β (test power) is 0.2 according to the statistical power analysis. If the value of n is increased, the effect size will be reduced, which can lead to statistically significant errors in each plane. Therefore, it may be difficult to challenge the usefulness of a positioning device based on this study’s results.

This study used a rapid prototyping model. In actual clinical cases, it may be difficult to use a positioning device when a fibula flap with soft tissue is used. However, we believe that if the design is changed to address the positional relationship between the device and the flap, a better surgical device can be manufactured. Depending on its position, the surgical device may interfere when a reconstruction plate is used; although, there should be no problems with the current design if a miniplate is used. Furthermore, the device design can be adjusted if a reconstruction plate is necessary.

Recently, the use of preoperative 3D surgical simulations for the manufacture of surgical guides has been on the rise [9, 10]. The use of surgical guides has also become popular in dental surgeries; in particular, dental implantations in mandibular reconstructions with the fibula can be planned preoperatively with a computer, and relevant surgical equipment can be manufactured for use in actual surgeries [11].

Essentially no clinical studies have described the use of the fibula positioning guide designed in the current study. However, Zheng et al. [12] reported a mandible reconstruction with cadaveric mandibles, where they used a fibular cutting guide and transferring guide, manufactured with preoperative 3D surgical simulation methods. Although a direct comparison with the present study was not possible, they reported an average error of 1.35 mm in the translation of fibular segments and an average error of 3.36° in the angular deviation of fibular segments. In contrast, in the present study, we found an error of about 16 mm in the condylar position, even when a fibula positioning guide was used. This discrepancy may be explained by differences in the experimental designs. Our experiments differed from real clinical cases, because we used models without temporomandibular joints or soft tissues. Moreover, the design of the positioning guide may have contributed to the results; the small supports for each fibula segment in the positioning guide, and the entire right mandibular area may have provided insufficient stability. This would have undermined the stability of the fibula positioning and fixation, which would ultimately generate a large error. Hence, there is a demand for studies that undertake optimizations in the size, position, and shape of the positioning guides to ensure sufficient stability in the mandibular area and fibula segments. This type of optimization could confer better experimental results.

The present study used two mini plates for each connection between fibula segments and between end segments and the mandible. This method for fixing the fibula segments could be modified as reconstruction plate for a better clinical result [13]. Recently, some studies have reported methods for manufacturing CAD/CAM reconstruction plates with 3D printers [14]. Schepers et al. [14] reported an average error of 3.0 mm in the deviation of fibular segments and an average error of 4.2° in the angular deviation of fibular segments in mandible reconstruction, when CAD/CAM reconstruction plates were used. That method enhanced the stabilization of the fibula segments during the positioning. However, for those methods, the metal plates must be manufactured with selective laser sintering and bio-appropriate metals, or it can be milled with a computerized, numerically controlled machine.

In the present study, all surgeries were conducted by a single surgeon. Some error may have been introduced, due to the surgeon’s level of proficiency. For instance, small inconsistencies in the contact angles or protrusions in the plating may have altered the abutment between the mandible and anterior fibula segment or between fibula segments. Also, the type of plate and the design of the surgical guides may have introduced errors.

If a reconstruction plate is used, the plate itself can act as a positioning guide for the fibula bone segment through pre-bending. In the present study, the positioning guide was used to secure the bone segment using a miniplate. The miniplate is fixed on the fibular lateral surface, so the positioning guide is presumed to be helpful. If a reconstruction plate is used, the design of the positioning guide can be adjusted to suit the position of the plate. Furthermore, for CAD/CAM reconstruction plates, which are manufactured to guide the position angle of the fibula bone segment according to the simulation outcome, a separate positioning guide may not be necessary.

The present study only sought to determine whether the fibula bone segment can be accurately positioned, according to the surgical simulation results, in cases of mandibular reconstruction using a fibula. The scope of the study is limited to the reconstruction of this hard tissue and the findings are not supported by actual clinical outcomes. If soft tissue is included with the fibula for the mandibular and facial reconstructions, there may be interference when positioning the cutting guide and positioning device on the fibula. Moreover, there may be difficulties in positioning the surgical device on the flap when thick muscle fibers are attached to the lateral surface of the fibula. A cutting guide can assist with accurate angles and positions, even with a small gap in between. However, a positioning device may incur greater error if soft tissues or thick muscles are attached to the fibula because the device is designed based the position of each fibula bone segment. Fixation plates, such as the miniplate or reconstruction plate, and screws limit the region of its placement. The usefulness of a positioning device may be different for each patient, as the fibula flap varies. Thus, CAD/CAM reconstruction plates may be a great alternative because they also guide the fibula bone segment to the accurate position and angle determined by surgical simulation.

Results of actual clinical cases are needed to evaluate the use of fibula positioning devices for facial reconstructions when soft tissues are included in the flap. In an actual surgery, the soft tissues may cause interference, so the positioning device must be designed in consideration of this. The positioning device was manufactured based on the simulation results using the patient’s actual CT data, so it is presumed there will be similar clinical outcomes as shown in the present study when only the bone segments are reconstructed using the fibula or when a fibula with little muscle fibers is used. In addition, future designs of the positioning device should be adjusted based on the soft tissue data.

In the present study, the condyle head was not fixed. Therefore, its position and movements may be different in an actual surgery, which is a limitation of this study. This study examined mandible reconstruction with subcondylar osteotomy, not a condylectomy, because mandible reconstruction accompanied by a condylectomy requires the fibula bone segment to be positioned on the glenoid fossa, but this distance and the three-dimensional position was not clearly identified. It is predicted that the error will be different in an actual surgery with condylectomy. Moreover, there may be a long-term error depending on the bone absorption in the distal end of the fibula bone segment, movement at the inner glenoid fossa, and changes in the glenoid fossa. These problems could be examined in an additional study on surgical planning with reference to the long-term prognosis. Although this study could not verify the stability of occlusion, we believe there will be stable occlusion if there is intermaxillary fixation with the side of the mandible that was not osteotomized, there is constant assessment, and implants and prosthetics are used when needed.

In the present study, only one surgeon manually fixed each bone segment, drilled, and inserted the screws simultaneously; therefore, there could have been error during the process of drilling and fixing each segment on the miniplate or in the manual holding of segments. However, this should not have induced a large experimental error because such errors can also be induced in the actual surgery. The outcomes of an actual surgery are presumed to be similar to that of this study if the condyle head is well fixed on the fossa and is not displaced significantly.

## Conclusions

This study aimed to examine the value of using a fibula segment positioning guide in mandibular reconstructions with fibula grafts. We found significant difference in the errors between reconstructions performed with or without the positioning guide, in the mean total 3D distance and the vertical error at the mesial point of the anterior fibula segment. Considering the CAD/CAM processes required for creating positioning devices, the positioning guide provided significant benefit over the use of a fibula cutting guide alone.

## References

[1] Liu XJ, Gui L, Mao C, Peng X, Yu GY (2009). Applying computer techniques in maxillofacial reconstruction using a fibula flap: a messenger and an evaluation method. J Craniofac Surg.

[2] Lethaus B, Kessler P, Boeckman R, Poort LJ, Tolba R (2010). Reconstruction of a maxillary defect with a fibula graft and titanium mesh using CAD/CAM techniques. Head Face Med.

[3] Ferri J, Piot B, Ruhin B, Mercier J (1997). Advantages and limitations of the fibula free flap in mandibular reconstruction. J Oral Maxillofac Surg.

[4] Foley BD, Thayer WP, Honeybrook A, McKenna S, Press S (2013). Mandibular reconstruction using computer-aided design and computer-aided manufacturing: an analysis of surgical results. J Oral Maxillofac Surg.

[5] Antony AK, Chen WF, Kolokythas A, Weimer KA, Cohen MN (2011). Use of virtual surgery and stereolithography-guided osteotomy for mandibular reconstruction with the free fibula. Plast Reconstr Surg.

[6] Mazzoni S, Marchetti C, Sgarzani R, Cipriani R, Scotti R, Ciocca L (2013). Prosthetically guided maxillofacial surgery: evaluation of the accuracy of a surgical guide and custom-made bone plate in oncology patients after mandibular reconstruction. Plast Reconstr Surg.

[7] Succo G, Berrone M, Battiston B, Tos P, Goia F, Appendino P (2015). Step-by-step surgical technique for mandibular reconstruction with fibular free flap: application of digital technology in virtual surgical planning. Eur Arch Otorhinolaryngol.

[8] Bell RB, Weimer KA, Dierks EJ, Buehler M, Lubek JE (2011). Computer planning and intraoperative navigation for palatomaxillary and mandibular reconstruction with fibular free flaps. J Oral Maxillofac Surg.

[9] Saad A, Winters R, Wise MW, Dupin CL, St Hilaire H (2013). Virtual surgical planning in complex composite maxillofacial reconstruction. Plast Reconstr Surg.

[10] Matros E, Santamaria E, Cordeiro PG (2013). Standardized templates for shaping the fibula free flap in mandible reconstruction. J Reconstr Microsurg.

[11] Zheng GS, Su YX, Liao GQ, Chen ZF, Wang L, Jiao PF (2012). Mandible reconstruction assisted by preoperative virtual surgical simulation. Oral Surg Oral Med Oral Pathol Oral Radiol.

[12] Zheng GS, Su YX, Liao GQ, Jiao PF, Liang LZ, Zhang SE (2012). Mandible reconstruction assisted by preoperative simulation and transferring templates: cadaveric study of accuracy. J Oral Maxillofac Surg.

[13] Azuma M, Yanagawa T, Ishibashi-Kanno N, Uchida F, Ito T, Yamagata K (2014). Mandibular reconstruction using plates prebent to fit rapid prototyping 3-dimensional printing models ameliorates contour deformity. Head Face Med.

[14] Schepers RH, Raghoebar GM, Vissink A, Stenekes MW, Kraeima J, Roodenburg JL (2015). Accuracy of fibula reconstruction using patient-specific CAD/CAM reconstruction plates and dental implants: a new modality for functional reconstruction of mandibular defects. J Craniomaxillofac Surg.

